# Non-pharmacological strategies for the management of Alzheimer's disease

**DOI:** 10.1590/1980-5764-DN-2025-0290

**Published:** 2026-08-21

**Authors:** Gunaro Lucas dos Santos de Oliveira, Amanda Gollo Bertollo

**Affiliations:** 1 Universidade Federal de Santa Catarina, Departamento de Farmacologia, Florianópolis SC, Brazil.

**Keywords:** Alzheimer Disease, Dementia, Therapeutics, Quality of Life, Complementary Therapies, Doença de Alzheimer, Demência, Terapêutica, Qualidade de Vida, Terapias Complementares

## Abstract

Alzheimer's disease (AD) is a multifactorial neurodegenerative disease affecting various cognitive domains, requiring a holistic and integrated therapeutic approach. The treatment of AD goes beyond pharmacological interventions, also necessitating non-pharmacological therapies that can act synergistically, both with each other and with emerging pharmacological treatments, including new anti-amyloid therapies. These therapies aim to improve patients’ quality of life and alleviate behavioral symptoms, such as agitation and depression, and delay disease progression. Current clinical and meta-analytic evidence suggests that these approaches can be effectively integrated with conventional treatment, offering a valuable alternative to enhance patients’ overall well-being. However, further research is needed to deepen the understanding of the ideal combination of the new anti-amyloid therapies, to address the limitations of current evidence, and to robustly establish their effectiveness in the clinical context of AD, aiming to generate a broader and more lasting impact on disease treatment. This narrative review aims to synthesize the evidence on diverse non-pharmacological therapies for managing AD and discuss their practical implications for clinical care.

## RESUMO

A doença de Alzheimer (DA) é uma doença neurodegenerativa multifatorial que afeta diversos domínios cognitivos, exigindo uma abordagem terapêutica holística e integrada. O tratamento da DA vai além das intervenções farmacológicas, necessitando também de terapias não farmacológicas que possam agir sinergicamente, tanto entre si quanto com os tratamentos farmacológicos emergentes, incluindo as novas terapias anti-amiloide. Essas terapias têm como objetivo melhorar a qualidade de vida dos pacientes e aliviar sintomas comportamentais, como agitação e depressão, além de retardar a progressão da doença. Evidências clínicas e meta-analíticas atuais sugerem que essas abordagens podem ser eficazmente integradas ao tratamento convencional, oferecendo uma alternativa valiosa para melhorar o bem-estar geral dos pacientes. No entanto, são necessárias mais pesquisas para aprofundar o entendimento da combinação ideal das novas terapias anti-amiloides, para abordar as limitações das evidências atuais, e estabelecer robustamente sua eficácia no contexto clínico da DA, com o objetivo de gerar um impacto mais amplo e duradouro no tratamento da doença. Esta revisão narrativa visa sintetizar as evidências sobre diversas terapias não farmacológicas para o manejo da DA e discutir suas implicações práticas para o cuidado clínico.

## INTRODUCTION

Alzheimer's disease (AD) is a progressive and debilitating neurodegenerative disease, representing the most prevalent cause of dementia, reflecting a striking challenge for healthcare professionals, researchers, caregivers, and patients^1^. The prevalence and incidence of AD continue to rise, particularly with the aging of the population, highlighting the urgent need for effective interventions to mitigate the burden of the disease^2^.

While current pharmacological treatments, including cholinesterase inhibitors (donepezil, rivastigmine, galantamine) and *N*-methyl-D-aspartate (NMDA) receptor antagonist (memantine) provide some symptomatic relief, they do not modify the underlying pathological progression of the disease^3^. However, recent advancements, including new anti-amyloid therapies like lecanemab and donanemab, offer the potential to slow disease progression by targeting core pathological mechanisms. In this evolving therapeutic landscape, non-pharmacological strategies are increasingly recognized as essential complementary components. They are vital for enhancing patients’ quality of life, managing behavioral and psychological symptoms of dementia (BPSD), and supporting functional independence, aspects not directly addressed by disease-modifying drugs^4-8^.

Robust evidence supports the efficacy of a diverse range of non-pharmacological interventions in the management of AD. These approaches are often first-line treatments for BPSD, improving mood and maintaining daily functioning. Psychosocial interventions, such as support groups and psychoeducation, are particularly effective for both people with dementia and their caregivers, notably reducing depression, anxiety, and caregiver burden^9,10^. Furthermore, multimodal strategies that combine cognitive, physical, and psychosocial elements show promise in stabilizing or improving cognition and functional independence^11^.

This narrative review aims to synthesize the evidence on specific, distinct non-pharmacological modalities, including physical exercise^12^, diet^13^, cognitive behavioral therapy (CBT)^14^, music therapy^15^, neurostimulation and cognitive training^16^, aromatherapy^17^, acupuncture^18^, and phytotherapy^19^. While general cognitive stimulation therapies are widely recognized, they are beyond the scope of this focused review. These approaches represent vital avenues for multidisciplinary clinical management of AD, exploring aspects not fully addressed by pharmacological options, and serve as viable alternatives for future research into multimodal interventions^20^.

## METHODS

This narrative review aimed to synthesize current evidence on diverse non-pharmacological interventions for Alzheimer's disease. To identify relevant literature, a comprehensive search was conducted across key electronic databases, including the United States National Library of Medicine (PubMed), Scopus, Embase, and Web of Science. Search terms encompassed "Alzheimer's disease" OR "dementia" combined with specific non-pharmacological interventions such as "physical exercise," "diet," "cognitive behavioral therapy," "music therapy," "neurostimulation," "aromatherapy," "acupuncture," and "phytotherapy". No formal language restrictions were applied, though the identified literature was predominantly in English. No specific time frame was used; however, preference was given to publications from the last ten years, with relevant older, seminal studies included as necessary to ensure comprehensive coverage of the literature. To organize the references, Zotero reference management software was used, following the recommendations of the Peer Review of Electronic Search Strategies (PRESS) guidelines^21^.

The initial article search, screening, and selection were performed independently by both authors, with discrepancies resolved through consensus. Articles were selected based on their relevance to the specific intervention being discussed and their contribution to understanding efficacy, proposed mechanisms, or clinical applicability. Preference was given to original research articles (e.g., clinical trials, observational studies), systematic reviews, and meta-analyses involving human subjects. Exclusion criteria included studies focusing solely on pharmacological treatments, those investigating other causes of dementia, and grey literature such as editorials and conference abstracts without complete publication. Given the nature of this narrative review, a formal risk-of-bias assessment was not conducted; however, emphasis was placed on synthesizing the highest level of evidence available (systematic reviews and randomized controlled trials — RCTs). The selection process focused on identifying a representative body of evidence that informs current understanding and highlights areas for future research within each modality. Eligibility and exclusion criteria are presented in Table 1.

**Table 1:** Eligibility criteria.

Inclusion criteria	
Publication date	Not applicable
Languages	English; Portuguese; Spanish.
Subjects	People diagnosed with Alzheimer's disease
Publication type	Randomized clinical trial; Non-randomized clinical trial; Observational study; Case study; Case-control study; Case report; Coort study; Systematic Reviews; Meta-analyses
Exclusion criteria	
Clinical studies in which other causes of dementia were investigated. Clinical studies in which only pharmacological disciplines were used as treatment.	

### Physical activity

Physical exercise has been shown to enhance cognitive performance, reduce behavioral symptoms, and promote overall well-being across various age groups and in different contexts of neurocognitive disorders, including AD. Regular physical exercise can also modulate mood and reduce anxiety, contributing to effects across multiple physiological systems in the body^12^.

Several studies suggest that physical activity can mitigate cognitive decline associated with aging and AD^22,23^. Chronic and moderate physical activity are associated with better cognitive performance and a lower risk of dementia in older adults^24^. These benefits are thought to arise from exercise-induced changes in brain structure and function, including slower rates of gray matter atrophy, preserved white matter integrity, maintained functional connectivity of brain networks, and enhanced synaptic integrity^25^.

Physical activity may act as a protective factor in AD development by restoring hippocampal function through the increased expression of brain-derived neurotrophic factor (BDNF) and other growth factors that promote neurogenesis, angiogenesis, and synaptic plasticity^26^. Resistance exercise and strength training can induce functional brain changes in the frontal lobe, improving executive functions, reducing white matter atrophy, and decreasing lesion volumes in white matter^27^.

Aerobic exercise training in middle-aged adults with a family history of AD elevates the biomarker Cathepsin B (CTSB) levels, which is positively associated with cognitive performance^28^. When performed at moderate to high intensity, exercise improves cardiorespiratory fitness in individuals with mild AD. It is related to improved cognition and reduced neuropsychiatric symptoms^29^. Moreover, aerobic exercise, such as moderate cycling performed thrice weekly, attenuated global cognitive decline in older adults with mild-to-moderate AD dementia^30^. It is important to note that some factors, such as exercise intensity, can influence the magnitude of exercise effects on physical and cognitive functions^31^. In addition to its biological and cognitive benefits, physical activity can also help to improve behavioral and psychological symptoms in subjects with AD. Older adults who are physically active are more likely to maintain cognitive function than their sedentary counterparts. Additionally, exercise interventions have improved cognitive outcomes in individuals already living with AD^23,24,29,32^.

Therefore, implementing lifestyle changes, such as regular physical exercise, may benefit the maintenance of individual cognitive health and significantly impact public health by reducing disease prevalence^33^.

### Diet

The diet for individuals with AD is essential for managing the disease and improving quality of life in hospitals and homes. Incorporating foods rich in antioxidants, omega-3 fatty acids, and other neuroprotective compounds may benefit brain function and potentially slow cognitive decline^13^. One dietary strategy is the ketogenic diet (KD), which has been shown to benefit cognitive functions in individuals with AD^34^. KD is a biochemically based diet that uses ketone bodies for brain metabolism. It has a low glycemic index and serves as an alternative glucose-oxidation source for neurons^34^, since glucose hypometabolism in the brain is a metabolic characteristic observed at early stages in AD^35,36^.

In the clinical study performed by Ota et al., 20 AD patients were randomized to receive a ketogenic formula (Ketonformula^®^) containing 20 g of medium-chain triglycerides (MCT) or an isocaloric placebo without MCT. Sixteen of the 20 patients completed the 12 weeks of treatment. The results revealed significant improvements in immediate logical memory and digit-symbol coding tests relative to baseline, and the authors concluded that chronic consumption of the formula may have beneficial effects on processing speed and verbal memory in individuals with AD^37^.

Reinforcing the use of MCT as a promising therapeutic approach due to being an excellent source of ketone bodies, the systematic review and meta-analysis of randomized clinical trials (RCT) by McKenzie et al. concluded that MCT consumption does not affect HDL, LDL, and total cholesterol levels^38^, making this dietary supplement slightly safe for future clinical trials involving individuals with AD, since many of the risk factors for AD involve lipid metabolism alterations, such as diabetes, obesity, hypertension, and high LDL cholesterol^39^.

In addition, Croteau et al. conducted a cross-sectional study comparing brain glucose and ketone metabolism in cognitively healthy elderly, those with mild cognitive impairment (MCI), and early-stage AD, concluding that ketogenic interventions can correct the glucose deficit in both MCI and AD^35^. A systematic review by Yusufov et al., which evaluated 65 studies published between 1997 and 2015 (with a total of 132,491 participants), concluded that 50 of these studies reported an association between diet and the incidence of AD, highlighting diet as a potentially modifiable risk factor for AD. There is also a significant association between AD and the Mediterranean diet (MeDi)^40^.

While studies suggest potential benefits of the ketogenic diet in AD, its highly restrictive nature presents significant practical challenges, particularly for individuals with cognitive impairment. Adherence can be difficult due to stringent dietary requirements, making it difficult for patients and caregivers to maintain over the long term. Furthermore, such a restrictive diet can lead to social isolation during meals or gatherings, potentially impacting the patient's quality of life and overall well-being. These factors are crucial considerations when evaluating the feasibility and widespread applicability of the ketogenic diet in clinical practice for AD^41,42^.

Among the most plausible diets in the clinical studies literature, the Mediterranean-DASH intervention for Neurodegenerative Delay (MIND) diet, which combines the MeDi and Dietary Approaches to Stop Hypertension (DASH) diets, is the most promising dietary program for treating AD and promoting quality of life in individuals^43^. In agreement, Levak et al. conducted a multimodal preventive trial with 93 participants with prodromal AD in different countries using nutritional guidance to assess the diet quality of these individuals. The results revealed that the group receiving multimodal intervention in lifestyle and medicinal diet improved their Healthy Diet Index (HDI) (p=0.042) and the Mediterranean Diet Adherence Score (MEDAS) (p=0.007) during the study. As part of the conclusion, the research revealed that nutrient intake in the control group decreased, whereas, in the treated groups, it remained unchanged^44^.

In this sense, two extensive reviews^45,46^ highlighted the importance of the ketogenic diet and the MIND diet as dietary strategies capable of reducing the progression of AD and lowering the risk of its development. Moreover, Neth et al., in a pilot study, revealed that the modified KD is associated with improved cerebrospinal fluid biomarker profiles^47^, while Nagpal et al. concluded that the modified Mediterranean-KD can modulate gut microbiota and short-chain fatty acids in association with AD markers in individuals with MCI^48^.

### Cognitive behavioral therapy

CBT has increasingly been used as a complementary strategy in the neuropsychological rehabilitation of patients with AD, particularly in its early stages. The main goal of CBT in this context is to alleviate symptoms of depression, anxiety, and behavioral changes, which affect about 90% of these individuals^49,50^. A study focused on neuropsychological rehabilitation and CBT in patients with AD showed that structured interventions targeting dysfunctional thoughts and behaviors can promote better coping strategies and increase autonomy in daily activities^51^.

Research highlights that CBT can enhance the effectiveness of cognitive rehabilitation by addressing cognitive and emotional symptoms. Patients who undergo CBT combined with cognitive rehabilitation show slower cognitive decline and better adherence to daily tasks. Integrating these therapies focuses on reinforcing cognitive strategies while addressing emotional symptoms such as anxiety and depression. Structured interventions that combine cognitive and behavioral components can significantly contribute to the patient's overall treatment plan^52^.

It is important to emphasize that CBT can effectively prevent or reduce symptoms of depression and anxiety by addressing these emotional and psychological symptoms early. CBT improves the patient's quality of life and can also slow the progression of cognitive decline associated with AD^53^.

In cases where depression coexists with AD, the application of CBT has been adapted to meet the cognitive limitations of such patients. A case study reported significant results when CBT was adapted to include shorter sessions, visual aids, and repetitive practice to reinforce learning. The treatment led to marked improvements in the patient's mood and a reduction in depressive symptoms over a series of sessions. Similarly, an open trial involving CBT for depression in patients with AD demonstrated that, even with cognitive decline, patients could engage with therapeutic techniques, leading to measurable emotional benefits and better symptom control^54^.

Furthermore, the CORDIAL program, which combines cognitive rehabilitation and CBT, was developed explicitly for patients with early-stage AD. Research findings showed that participants in this program experienced cognitive improvements and greater emotional resilience, resulting in better adaptation as the disease progressed. The multidisciplinary nature of this program highlights the importance of early and comprehensive interventions that address cognitive and emotional challenges, providing a more holistic approach to treatment^14^.

Finally, CBT plays a crucial role in preventing AD by addressing risk factors such as physical inactivity, poor diet, sleep disturbances, and lack of cognitive stimulation. Managing these factors through structured interventions can reduce the likelihood of disease development^55^.

### Music therapy

Research demonstrates that music therapy addresses the behavioral, cognitive, and emotional challenges of AD^56-59^.

It is important to distinguish between general music activities, such as listening to music or singing in a group, and formally structured music therapy. While both can offer benefits, true music therapy is a clinical and evidence-based intervention delivered by a credentialed music therapist to achieve individualized non-musical goals within a therapeutic relationship. Unlike casual music engagement, music therapy involves a structured process, including assessment, treatment planning, and evaluation, with a focus on specific therapeutic outcomes^60,61^.

Structured musical activities stimulate brain areas essential for memory and attention, evoking emotional responses that enhance cognitive performance. Regular sessions have been shown to improve mental functions and address cognitive decline while simultaneously supporting the emotional and social well-being of AD patients. Crucially, music therapy has also demonstrated significant efficacy in managing BPSD, often leading to a reduction in agitation, aggression, and anxiety, and an improvement in mood and sleep patterns^62,63^. Despite some variability in how music therapy is applied across studies, its potential as an effective intervention for AD is increasingly recognized and recommended in clinical practice^23^.

The biological mechanisms underlying the benefits of music therapy in AD are multifaceted and encompass various neurological, neurochemical, and psychosocial processes. At the neurological level, research has shown that music therapy can activate multiple brain regions, including the auditory cortex, limbic system, and frontal lobe, commonly affected by the progressive neurodegeneration observed in AD^64^. Activating these brain regions may enhance cognitive function, improve emotional regulation, and increase social engagement.

From a neurochemical perspective, music therapy has been associated with modulating neurotransmitter and hormone release, including dopamine, serotonin, and oxytocin. These neurotransmitters regulate mood, social bonding, and stress reduction^65,66^. Studies have demonstrated that music can increase dopamine production, a neurotransmitter associated with pleasure and reward. This may contribute to improved mood and reduced apathy in individuals with AD^67^.

In addition to the neurological and neurochemical mechanisms, music therapy has also shown significant psychosocial benefits for individuals with AD. It facilitates communication and interpersonal processes, enhances emotional expression, and promotes social connection^68^. The music therapy literature has recognized the importance of this musical element, placing it at the core of each individual's intersubjective development^68,69^.

### Neurostimulation

The brain stimulation techniques most studied for the treatment of dementia and MCI include transcranial direct current stimulation (tDCS) and transcranial magnetic stimulation (TMS/rTMS). These techniques are often tested in combination with cognitive training in clinical trials to achieve clinically meaningful improvements in cognitive function.

One study utilized a combination of tDCS (20 minutes/session) with computerized cognitive training (CCT) over three weeks, totaling nine sessions, compared to a tDCS-sham group and a group receiving only CCT to treat individuals with MCI. The results showed significant improvement in cognitive performance on the Mini-Mental State Examination (MMSE) and processing and attention^70^. These improvements, while encouraging, often represent modest but statistically significant gains in specific cognitive domains.

Another study tested the combination of rTMS and cognitive training (COG) over four weeks with one-hour/day sessions, followed by four weeks of maintenance, in individuals with AD. This combination resulted in improved Alzheimer's Disease Assessment Scale — Cognitive Subscale (ADAS-Cog) and Montreal Cognitive Assessment (MoCA) scores, demonstrating a positive impact on cognitive function across multiple brain regions^71^.

Similarly, a study tested the combination of TMS and CCT over 30 sessions over six weeks to treat AD. The results revealed significant cognitive improvement through ADAS-Cog, with the active treatment group showing a mean improvement of 4.4 points (from 21.7 to 17.3), indicating a clinically relevant benefit^72^.

A variation of TMS, rTMS combined with CT, was used in patients with AD. The sessions lasted one hour/day, five days/week, for six weeks, and the technique was maintained every two weeks for the following three months after the intervention. Significant improvement in overall cognitive performance measured by ADAS-Cog was observed in the treated group, suggesting a sustained positive effect^73^.

Another study used a combination of rTMS (10 Hz) and CT across 30 sessions over six weeks in patients with AD and reported improved ADAS-Cog scores, with effects significantly superior on the orientation subscale compared with the sham group, highlighting a targeted cognitive benefit^74^.

These results corroborate the conclusions of systematic reviews and meta-analyses, which highlight that tDCS and TMS/rTMS techniques have significant clinical value for treating patients with AD and MCI, especially patients who show no improvement in neurocognitive tests despite using medications to control cognitive decline in dementia^75,76^. However, although evidence is growing, these techniques are still largely considered investigational or adjunctive, and the magnitude and long-term durability of their effects require further robust, large-scale, and standardized clinical trials to establish their definitive role in routine clinical practice.

### Aromatherapy

Aromatherapy uses plants’ essential oils (EOs) to promote therapeutic effects in various disorders and conditions, including AD^77^. EOs act on the limbic system after entering the bloodstream via inhalation (via the olfactory nerve) or application to the skin, directly affecting cognition^78^ through mechanisms related to the oxidative stress hypothesis and the cholinergic hypothesis in the pathophysiology of AD^79^.

In a crossover clinical trial involving 28 volunteers, 17 of whom were diagnosed with AD, rosemary and lemon essential oils were used in the morning, and lavender and orange essential oils in the evening for 28 days. The researchers used four validated assessment scales to track the effects of the intervention. Two of the scales reported significant improvement for all volunteers, and in particular, volunteers with AD showed substantial improvement in the total scores on one of the scales (Japanese version of the Gottfries, Brane, and Steen Scale — GBSS-JA-13; item 13 on the scale assesses abstract function). Additionally, routine laboratory tests did not reveal significant changes, suggesting no adverse effects from the use of the oils. Moreover, another test showed that caregivers’ scores did not affect the evaluation scales, reducing bias in the intervention results^80^.

In another 8-week clinical trial involving 36 volunteers, the effects of olfactory nerve stimulation via inhalation of an aromatic extract containing cedar essential oil were evaluated. The exposure improved the behavioral and psychological symptoms of AD in these participants, assessed by validated tests^81^. A double-blind, placebo-controlled clinical trial involving 72 patients used *Melissa officinalis* essential oil, applied topically to different body areas twice daily for four weeks. It reported significant improvement in the Cohen-Mansfield Agitation Inventory (CMAI) scores, where 60% of patients in the treated group showed a 30% reduction in the CMAI score, compared to only 14% of patients in the placebo group (sunflower oil), demonstrating the benefits of *M. officinalis* essential oil for agitation in individuals with severe dementia^82^.

Despite promising results from various clinical trials of aromatherapy for dementia, it is essential to exercise caution in interpreting both the data and the methodology. The primary limitation resides in the methodological diversity and the hierarchy of evidence across the studies. Although the trial using *Melissa officinalis* achieved the gold standard (double-blind and placebo-controlled), most research still relies on relatively small samples (such as the studies involving 28 and 36 volunteers) and designs that, despite being controlled or crossover, may not have rigorously implemented blinding, an inherent challenge for interventions with a strong olfactory component. The lack of robust randomization or blinding increases the risk of bias, whether from the placebo effect or from the expectations of researchers and caregivers, even though some studies have shown that caregivers do not affect the assessment. Thus, to validate the efficacy of essential oils, more large-scale RCTs are required to explicitly compare different oils, dosages, and routes of administration, to establish a standardized therapeutic protocol supported by high-level evidence.

### Acupuncture

Preliminary evidence suggests that acupuncture may help to alleviate symptoms of agitation^18^ and sleep disorders^83^, which are common in individuals with AD, by promoting relaxation and maintaining blood flow.

Acupuncture may improve mood and sleep by modulating dopaminergic and serotonergic systems and potentially reducing beta-amyloid deposition, a key pathophysiological mechanism of AD^83,84^.

Studies indicate that acupuncture may affect the cholinergic system, which is impaired in AD^85^, by increasing acetylcholine levels and enhancing the activity of choline acetyltransferase (ChAT) and acetylcholinesterase (AChE), thereby enhancing cholinergic transmission in AD models^86-88^.

Moreover, acupuncture has also been shown to have the potential to reduce oxidative stress by activating the Nrf2/ARE pathway, which is crucial for regulating redox balance^86,89,90^. It contributes to reducing neuroinflammation by decreasing the expression of pro-inflammatory cytokines (IL-1β, TNF-α, IL-6) and increasing anti-inflammatory cytokines (IL-4, IL-10) in animal models of AD, thereby improving cognitive function^87,90,91^.

Studies in animal models of AD also suggest that acupuncture can enhance learning and memory capacity^92^, and cognition, increase synaptic plasticity, and promote neuroprotection^93^. Furthermore, another effect of acupuncture identified in animal models of AD is the reduction of neuronal apoptosis by negatively regulating genes and proteins involved in apoptosis, thereby improving neuronal survival and cognitive function^90^. These preliminary findings underscore the importance of rigorously testing the validity of this therapy in clinical trials.

Although the literature points to several promising mechanisms for its treatment of AD, including modulation of neurotransmitters (such as serotonin and dopamine), reduction of oxidative stress, decreased neuroinflammation, and enhanced neuroprotection in animal models, high-level clinical evidence remains scarce. The main gap lies in the translatability of these preliminary findings from animal models to clinical practice, as *in vivo* efficacy must be rigorously validated in robust clinical trials. The complexity of the intervention also poses critical methodological challenges, including difficulties in effectively implementing blinding (for both the patient and the therapist), which increase the risk of performance bias and the placebo effect. Therefore, for acupuncture to be considered a validated therapy, more high-quality RCTs are essential to establish standardized protocols for points and frequency, overcome blinding bias, and confirm the neurological benefits observed in the laboratory.

### Phytotherapy

Clinically prescribed for their phytochemical content, herbal preparations are valuable therapeutic resources for AD management supported by numerous studies^94,95^.

A randomized, double-blind, placebo-controlled 4-month clinical trial used *Melissa officinalis* extract at a fixed dose of 60 drops per day, administered orally, to evaluate its effects in participants (20 treated vs. 15 placebo) with mild-to-moderate AD. The results revealed that the extract significantly improved cognitive function, as assessed by the ADAS-Cog, compared to the placebo. Moreover, no differences in side effects were observed between the two groups, except for agitation, which was more common in the placebo group^96^.

*Curcuma longa*, which contains the phytochemical curcumin, is one of the most studied phytotherapeutics for neurodegenerative diseases. In a four-week clinical trial, blood and saliva were collected from volunteers (19 interventions vs. 19 placebo) without AD. Lipid-based curcumin supplementation (80 mg/day) promoted several beneficial changes, including reduced plasma beta-amyloid peptide concentrations^97^.

Another randomized clinical trial used dietary curcumin supplementation (180 mg/day) for 12 weeks in adults at high risk for insulin resistance (IR) and AD. The results showed that supplementation significantly reduced circulating glycogen synthase kinase-3 beta (GSK-3β) levels and islet amyloid polypeptide (IAPP) levels, and reduced IR, compared with the placebo group. These findings suggest a new mechanism by which curcumin may influence markers of IR and AD, as defective insulin signaling in brain tissue promotes the accumulation of Tau protein and beta-amyloid peptides, linking the pathophysiology of such diseases. These correlations are highly significant, as the prevalence of IR is rising, with over 463 million cases of diabetes worldwide, of which type 2 diabetes (T2D) accounts for more than 90% of these cases^98^.

Although phytotherapy is recognized as a valuable therapeutic resource, several clinical trials attest to the benefits of compounds such as *Melissa officinalis* and *Curcuma longa* (curcumin) in improving cognitive function and modulating pathophysiological markers of AD. However, crucial limitations persist. The main one lies in the variety of study designs and samples. While the use of *Melissa officinalis* was supported by high-quality evidence (a randomized, double-blind, placebo-controlled trial), the cited curcumin studies focus, for example, on individuals without an AD diagnosis or in high-risk populations for IR. These focuses, although essential for elucidating mechanisms (such as the reduction of plasma beta-amyloid peptides and modulation of GSK-3β), do not prove the clinical efficacy of curcumin in the direct treatment of established dementia. The heterogeneity of interventions, ranging from standardized extracts to lipid-based supplements (for curcumin), and the variation in dosages and treatment periods complicate the standardization of a single therapeutic protocol and the direct comparison of results. Therefore, more long-term RCTs focusing on patients with established AD are necessary to consolidate phytotherapy as a definitive treatment.

## DISCUSSION

The non-pharmacological strategies discussed in this study demonstrate significant potential for managing AD by improving cognitive, behavioral, and functional symptoms. Combining these therapies with pharmacological approaches offers a promising avenue for a more effective and holistic intervention that addresses the disease's multifactorial complexity. Table 2 provides a comparative overview of the therapeutic approaches considered in this narrative review.

**Table 2:** Comparison of non-pharmacological strategies for Alzheimer's disease.

Intervention	Proposed mechanism of action	Main clinical evidence	Reported effects	Level of recommendation
Physical exercise	↑ BDNF, neurogenesis, vascularization	Clinical trials, meta-analyses	Improved cognition, ↓ BPSD	High
Diet (MIND, ketogenic)	↑ ketone bodies, ↓ inflammation	Observational studies, RCTs	Stabilizes or improves cognition	Moderate/High
CBT	Cognitive and emotional restructuring	Clinical trials; open studies	↓ anxiety, ↑ adherence to routines	Moderate
Music Therapy	Limbic and dopaminergic activation	RCTs and longitudinal studies	↓ agitation, ↑ social interaction	Moderate
Neurostimulation	Cortical modulation	Sham-controlled RCTs	↑ memory and executive function	Moderate
Aromatherapy	Olfactory stimulation, ↓ oxidative stress	Small RCTs	↓ agitation, behavioral improvement	Low/Promising
Acupuncture	Inflammatory and cholinergic regulation	Animal and human studies	↑ cognition, ↓ behavioral symptoms	Low/Promising
Phytotherapy	Anti-inflammatory and antioxidant effects	RCTs with specific extracts	↓ agitation, ↑ cognitive performance	Moderate

Abbreviations: BDNF, Brain-Derived Neurotrophic Factor; BPSD, Behavioral and Psychological Symptoms of Dementia; RCT, Randomized Controlled Trial; CBT, Cognitive Behavioral Therapy.

Notes:

↑ Increased / improved;

↓ Decreased / reduced.

**Figure 1:**
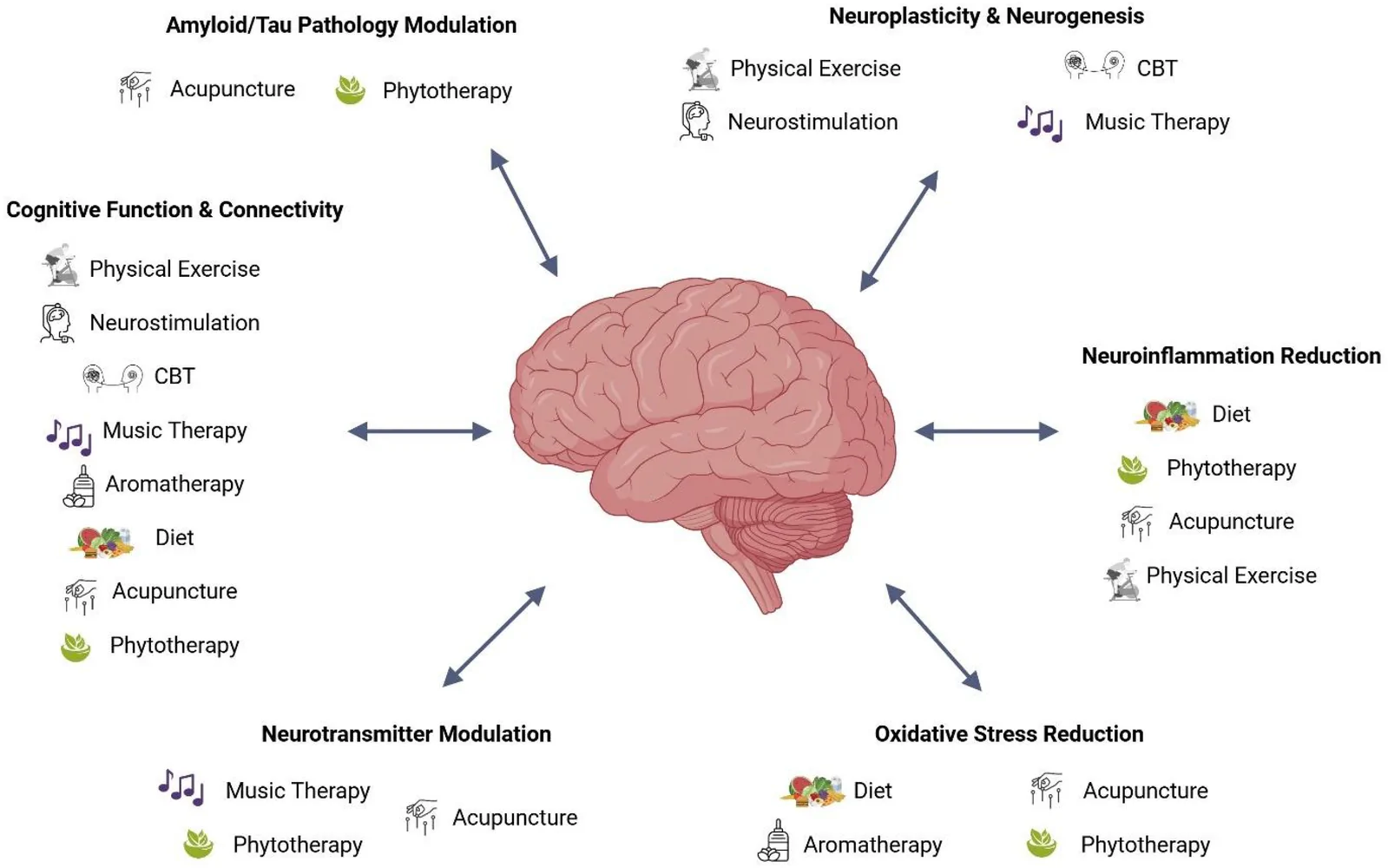
Key brain pathways modulated by non-pharmacological interventions in Alzheimer's disease.

Note: This figure illustrates the primary neurological pathways and processes in the brain that areimplicated in Alzheimer's disease and influenced by various non-pharmacological interventions. Each pathway lists the specific interventions that have demonstrated effects on that particular mechanism or function, highlighting the multifaceted and convergent nature of these therapeutic approaches.

Abbreviation: CBT, Cognitive Behavioral Therapy.

Despite the encouraging potential of these interventions, a critical analysis of the current evidence reveals several common methodological limitations. Many studies are characterized by relatively small sample sizes, which can limit the statistical power and generalizability of findings. Furthermore, a substantial number of investigations are not RCTs, increasing the potential for bias. Significant heterogeneity exists in intervention protocols, durations, and outcome measures across studies, making direct comparisons and the establishment of standardized guidelines challenging. Moreover, the lack of long-term follow-up in many studies limits our understanding of the sustained benefits and real-world durability of these effects^2,32^. The diverse effects of these interventions on key brain pathways and their potential mechanisms are visually summarized in Figure 1.

Beyond methodological considerations, the practical implementation and long-term adherence to non-pharmacological therapies in AD patients present considerable challenges. These include ensuring patient adherence, particularly as cognitive impairment progresses, and the availability of specialized resources and trained personnel. The crucial involvement and support of family caregivers are paramount for facilitating patient participation and ensuring sustained adherence, underscoring the need to consider caregiver burden and provide adequate support structures in any intervention strategy^49,50^.

A comparative analysis of the evidence (Table 2) reveals that non-pharmacological strategies with strong clinical support, such as physical exercise and dietary approaches (e.g., the MIND diet), often target multiple pathophysiological pathways of AD. These interventions not only promote cognitive benefits but also improve mood, cardiovascular health, and metabolic function, areas frequently comorbid with AD. Complementary therapies like CBT and music therapy demonstrate substantial promise in addressing specific symptoms such as depression, anxiety, or BPSD, as well as enhancing social interaction.

On the other hand, interventions such as acupuncture, aromatherapy, and phytotherapy, while gaining interest and often being culturally accepted, are currently supported by more limited and heterogeneous clinical evidence. These approaches may serve as valuable adjuncts, particularly in palliative care settings or among patients with poor tolerance to pharmacological treatments. However, their implementation should be based on updated clinical guidelines and tailored to individual needs. From this perspective, an integrative model is proposed, structured around three therapeutic pillars: core interventions with strong evidence (e.g., physical exercise, MIND diet) serve as the foundation of care; complementary therapies (e.g., music therapy, CBT) are tailored to specific symptoms such as depression, anxiety, or social withdrawal; and adjuvant symptomatic support (e.g., aromatherapy, acupuncture, phytotherapy) aims to enhance comfort, reduce neuropsychiatric symptoms, and support caregiver well-being. This integrative approach recognizes the complexity of AD and the necessity of combining individualized, multimodal interventions with interdisciplinary collaboration to optimize outcomes in real-world clinical practice.

The proposal for a non-pharmacological and integrative intervention model must, however, be analyzed from the perspective of implementation feasibility across diverse real-world settings, especially those with limited financial and human resources. While combining therapies optimizes clinical outcomes, the associated costs, the need for multiple specialized professionals (such as physical therapists, nutritionists, and music therapists), and the scheduling logistics can create significant barriers to sustained adherence and equitable access. In resource-scarce environments, prioritization must focus on interventions with the highest cost-effectiveness and those that can be adapted and sustained more readily. This includes physical exercise and dietary approaches, which generally require less specialized equipment and can be facilitated through basic caregiver and volunteer training and by leveraging existing community resources.

Finally, successful integration of non-pharmacological strategies into AD management requires an individualized approach that accounts for disease stage, the patient's clinical conditions, and personal preferences. The consistent involvement of a multidisciplinary team, alongside the crucial support of family caregivers, is essential for implementing these interventions effectively and sustainably.

In conclusion, this review highlights that non-pharmacological strategies are crucial in managing AD and significantly benefit patients and caregivers. Therapies such as physical exercise, a balanced diet, CBT, music therapy, neurostimulation, aromatherapy, acupuncture, and phytotherapy complement one another and contribute to improved quality of life and overall functioning in affected individuals.

Despite promising results, further rigorous clinical studies are necessary to establish implementation guidelines and confirm the long-term efficacy of these interventions. Moreover, a multidisciplinary, individualized approach should be prioritized, taking into account each patient's needs and the synergy among therapies.

In practical terms, clinical care teams are encouraged to prioritize interventions with the most substantial evidence, such as physical activity programs and dietary modifications (e.g., the MIND diet), as core strategies, integrating them early in the disease trajectory. Complementary approaches like music therapy and CBT should be selectively applied based on neuropsychiatric symptoms and functional decline. Adjuvant therapies such as aromatherapy, acupuncture, and phytotherapy may be used to support symptom relief and enhance quality of life, particularly in moderate to advanced stages, provided safety and cultural acceptability are ensured.

Future research should focus on:

- the development of standardized protocols for combined (multimodal) non-pharmacological interventions;
- large-scale, blinded, multicenter trials assessing real-world effectiveness;
- cost-effectiveness studies to support incorporation into public health systems; and
- investigations into the biological mechanisms that underpin the observed clinical benefits.

Special attention should also be given to personalizing these interventions based on patient profiles, disease stage, and caregiver support structures.

By addressing these clinical and scientific priorities, non-pharmacological therapies can be more effectively translated into evidence-based guidelines and integrated care pathways, ultimately improving the lives of people living with AD and reducing the burden on caregivers and healthcare systems.

Given the significant impact of AD on public health, promoting the use of non-pharmacological strategies represents an essential advance for comprehensive and humane care, offering safe and effective alternatives for managing this complex and debilitating condition.

## Data Availability

No new data were generated or analyzed in this study.
